# Activation of transient receptor potential ankyrin 1 induces CGRP release from spinal cord synaptosomes

**DOI:** 10.1002/prp2.191

**Published:** 2015-10-25

**Authors:** Talisia Quallo, Clive Gentry, Stuart Bevan, Lisa M. Broad, Adrian J. Mogg

**Affiliations:** ^1^ Wolfson Centre for Age Related DiseasesKing's College LondonLondonSE1 1ULUnited Kingdom; ^2^ Neuroscience Research DivisionLilly Research CentreEli Lilly & Co. LtdWindleshamUnited Kingdom

**Keywords:** CGRP, spinal cord, synaptosomes, TRP channels, TRPA1

## Abstract

Transient receptor potential ankyrin 1 (TRPA1) is a sensor of nociceptive stimuli, expressed predominantly in a subpopulation of peptidergic sensory neurons which co‐express the noxious heat‐sensor transient receptor potential vanilloid 1. In this study, we describe a spinal cord synaptosome‐calcitonin gene‐related peptide (CGRP) release assay for examining activation of TRPA1 natively expressed on the central terminals of dorsal root ganglion neurons. We have shown for the first time that activation of TRPA1 channels expressed on spinal cord synaptosomes by a selection of agonists evokes a concentration‐dependent release of CGRP which is inhibited by TRPA1 antagonists. In addition, our results demonstrate that depolarization of spinal cord synaptosomes by a high concentration of KCl induces CGRP release via a T‐type calcium channel‐dependent mechanism whilst TRPA1‐induced CGRP release functions independently of voltage‐gated calcium channel activation. Finally, we have shown that pre‐treatment of synaptosomes with the opioid agonist, morphine, results in a reduction of depolarization‐induced CGRP release. This study has demonstrated the use of a dorsal spinal cord homogenate assay for investigation of natively expressed TRPA1 channels and for modulation of depolarizing stimuli at the level of the dorsal spinal cord.

**Abbreviations**
ANOVA: anaylsis of variance
CGRP: calcitonin gene‐related peptide
ELISA: enzyme‐linked immunosorbent assay
eEPSC: evoked excitatory postsynaptic current
mEPSC: miniature excitatory postsynaptic current
TRPA1: transient receptor potential ankyrin 1
TRPV1: transient receptor potential vanilloid 1
VGCC: voltage‐gated calcium channel

## Introduction

Neuropeptides (e.g., calcitonin gene‐related peptide [CGRP] and Substance P) released from primary afferent fibres relay nociceptive information in the spinal cord and play an important role in pain hypersensitivity (Gangadharan and Kuner 2013). Transient receptor potential ankyrin 1 (TRPA1) is expressed in small to medium diameter neurons which predominantly co‐express the noxious heat‐sensor transient receptor potential vanilloid 1 (TRPV1), but is also expressed by sensory neurons which are TRPV1‐negative (Zygmunt and Högestätt 2014). Similarly, TRPA1 is found in both peptidergic, IB4‐negative, and IB4‐positive neuron populations. The role TRPA1 plays in sensing chemical and thermal stimuli in the periphery is well characterized (Story et al. 2003; Bandell et al. 2004; Andersson et al. 2008; Karashima et al. 2009, 2009), yet the function of TRPA1 channels expressed on the postsynaptic membranes of primary afferent fibers, remains ambiguous (Kim et al. 2010). TRPA1 has been suggested to play a role in spinal processing of nociceptive input. Activation of TRPA1 expressed on central terminals of primary afferent fibers has been shown to increase the frequency of miniature excitatory postsynaptic currents (mEPSCs) and diminish the amplitude of eEPSCs in subpopulations of laminae I and II neurons (Wrigley et al. 2009). In addition, it has been shown that intrathecal administration of TRPA1 agonists can interrupt nociceptive transmission by reducing voltage‐gated calcium and sodium currents (Andersson et al. 2011). There are limited high‐throughput methodologies for studying native channels expressed on central terminals of primary afferent fibers and the mechanisms by which these channels influence neurotransmitter release. A greater understanding of these mechanisms could facilitate the discovery of novel anti‐nociceptive therapies. In this study, we describe the adaptation of a spinal cord synaptosome‐release assay (Mogg et al. 2013) to study native, spinal TRPA1 channels. We have measured CGRP release from rat spinal cord synaptosomes in response to TRPA1 agonists and membrane depolarization and have examined the effect of TRPA1 antagonists, voltage‐gated calcium channel (VGCC) inhibitors and opioid receptor ligands.

## Materials and Methods

### Materials

Some experiments were completed with Tyrode's salts with HEPES buffer (041‐95897M; Invitrogen, Thermo Fisher Scientific, Waltham, MA, USA) + 10 *μ*mol/L thiorphan (to prevent CGRP breakdown by endogenous proteases) but the majority of the experiments were conducted in HEPES buffered Tyrode's solution, HBTS (136 mmol/L NaCl, 2.7 mmol/L KCl, 1.8 mmol/L CaCl_2_, 1.05 mmol/L MgCl_2_, 5 mmol/L glucose, 10 mmol/L HEPES and 335 *μ*mol/L NaH_2_PO_4_)* *+ 10 *μ*mol/L thiorphan buffered to pH 7.4 (NaOH). For Ca^2+^‐free experiments CaCl_2_ was omitted from the solution and 1 mmol/L EGTA was added.

AITC (allyl‐isothiocyanate), cinnamaldehyde, morphine and naloxone were obtained from Sigma‐Aldrich (St Louis, MO). Nifedipine, mibefradil dihydrochloride, *ω*‐conotoxin MVIIC were from Tocris Bioscience (Bristol, UK). 4‐HNE (4‐hydroxynonenal) and 4‐ONE (4‐oxo‐2‐nonenal) were obtained from Cayman Chemical (Ann Arbour, MI, USA). AP18 was from Maybridge (Tintagel, Cornwall, UK). All master stock solutions were aliquotted and stored at −20°C. Dilutions from these aliquots into HBTS were made daily for their use in experiments.

### Methodology for 96 well CGRP release assay

Adult, male, Sprague Dawley rats (~250–500 g) were killed by exposure to a rising concentration of CO_2_ or by cervical dislocation. The lumbar portion of the spinal cord was then dissected (in some experiments the dorsal and ventral regions of the cord were separated by dissection), weighed and placed in a Sterilin tube containing ice‐cold HBTS buffer without thiorphan. The cord was homogenized using approximately 12–15 strokes of a glass‐Teflon hand homogenizer. The homogenate was then centrifuged for 1 min at 168*g* in a bench‐top centrifuge and the supernatant carefully removed and reconstituted at a final concentration of 4 mg·mL^−1^ in HBTS. 100 *μ*L of the homogenate solution was placed in each well of a 96‐well filter plate (type MSBVN1B50, Millipore, Billerica, MA, USA) using a multichannel pipette. The use of filter plates in these experiments allowed the solutions bathing the synaptosomes to be easily removed. The filter plate containing the homogenate was incubated at 37°C in a humidified incubator gassed with 5% CO_2_ in air for 20 min prior to compound addition. Following the 20 min incubation period, the filter plate containing the homogenate was removed from the incubator and the buffer in the wells filtered to waste using a vacuum manifold. Extracellular solutions either in the presence or absence of antagonists or modulators were added to the wells of the filter plate (100 *μ*L per well) and the filter plate was incubated for 10 min at 37°C. The contents of the filter plate were then filtered to waste using a vacuum manifold. Extracellular solutions containing agonists or KCl (either in the presence or absence of antagonists or modulators) were then added to the wells of the filter plate (100 *μ*L per well) and incubated for a further 10 min at 37°C. Following the stimulation period, solutions contained within the wells of the filter plate were rapidly transferred to the wells of an enzyme‐linked immunosorbent assay (ELISA) CGRP immunoplate (SPIbio, now Bertin Pharma, Montigny le Bretonneux, France) containing anti‐CGRP‐AChE tracer (100 *μ*L per well) by centrifugal force (200*g*). The immunoplate was then covered with plastic film and incubated at 4°C for 16–20 h. The following day, the immunoplate was washed four times and Ellman's reagent was added to the wells of the plate (200 *μ*L). The plate was left at room temperature to develop in the dark for 45 min. The absorbance of each well was then measured at 405 nm using a 96‐well plate spectrophotometer (Molecular Devices, Sunnyvale, CA, USA).

### Data analysis

Raw absorbance values were imported into Excel (Microsoft) for further analysis and then into Origin Pro, version 9.1 (OriginLab Corporation, Northampton, MA, USA) for graphical representation of the results. The amount of CGRP released was reported as a percentage over basal release (the amount of CGRP released upon addition of HBTS) or as a percentage of the 40 mmol/L KCl‐induced release. Release stimulated in the presence of an antagonist or modulator was normalized to an appropriate antagonist/modulator basal release where possible. Where stated, *n* refers to the number of independent experiments performed using spinal cord tissue from different animals. In each individual experiment a minimum of 4 replicate samples per treatment were used.

### Statistics

Normality of data was tested using the Shapiro–Wilk Test and homogeneity of variances tested using Levene's test. Differences in means between two groups were analyzed using an independent samples *t*‐test. Differences in means between three groups or more were analyzed using a one‐way analysis of variance (ANOVA), followed by a Tukey's HSD post hoc test (for datasets with equal variances) or a Dunnett's T3 post hoc test (for datasets with unequal variances). All statistical analyses were made using IBM SPSS statistics, version 22 (IBM, Armonk, NY, USA).

## Results

### Activation of TRPA1 evokes CGRP Release from spinal cord homogenate

**Figure 1:**
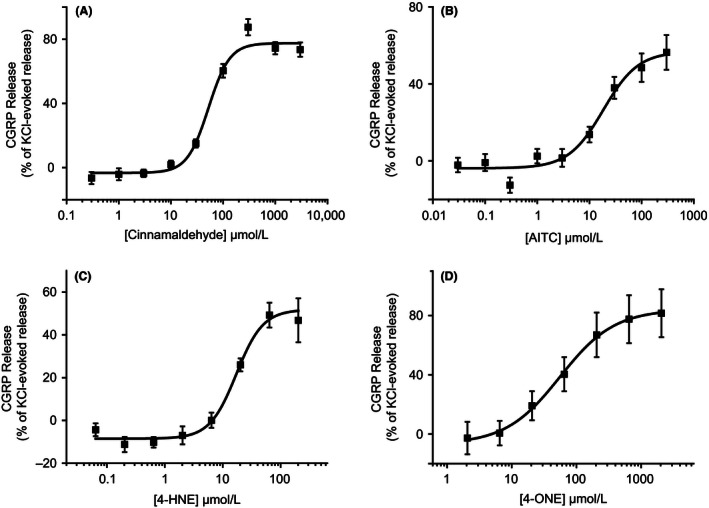
Activation of transient receptor potential ankyrin 1 (TRPA1) evokes calcitonin gene‐related peptide (CGRP) release. The release of CGRP from rat spinal cord homogenate was measured by ELISA. Application of TRPA1 agonists evoked concentration‐dependent increases in CGRP release from spinal cord homogenate. (A) Cinnamaldehyde‐induced CGRP release with an EC_50_ value of 54.78 ± 5.84 *μ*mol/L (mean ± SEM; *n* = 10). (B) AITC evoked CGRP release with an EC_50_ value of 17.08 ± 0.77 *μ*mol/L (mean ± SEM;* n* = 3). (C) The endogenous TRPA1 agonist 4‐HNE stimulated CGRP release with an EC_50_ value of 17.39 ± 0.68 *μ*mol/L (mean ± SEM;* n* = 3). (D) Another endogenous TRPA1 agonist, 4‐ONE, also induced CGRP release with an EC_50_ value of 63.80 ± 4.26 *μ*mol/L (mean ± SEM;* n* = 3). Representative plots shown, data points are the mean normalized release (% of 40 mmol/L KCl‐induced release) of quadruplicate wells ± SEM.

In order to examine whether activation of TRPA1 evokes CGRP release from rat spinal cord synaptosomes, spinal cord homogenate was stimulated with TRPA1 agonists and the resulting CGRP release examined by ELISA. Stimulation of rat spinal cord homogenate by the pungent TRPA1 agonist, cinnamaldehyde, evoked a concentration‐dependent increase in CGRP release with a mean EC_50_ value of 54.79 ± 5.84 *μ*mol/L (mean ± SEM; *n* = 10 experiments; Fig. 1A). Activation by allyl‐isothiocyanate (AITC) also evoked CGRP release with an EC_50_ value of 17.08 ± 0.77 *μ*mol/L (mean ± SEM; *n* = 3 experiments; Fig. 1B). CGRP release was also evoked by electrophilic, endogenous agonists of TRPA1. 4‐HNE and 4‐ONE evoked CGRP release from spinal cord homogenate with EC_50_ values of 17.39 ± 0.68 *μ*mol/L and 63.80 ± 4.26 *μ*mol/L, respectively (mean ± SEM; *n* = 3 experiments; Fig. 1C and D).

**Figure 2:**
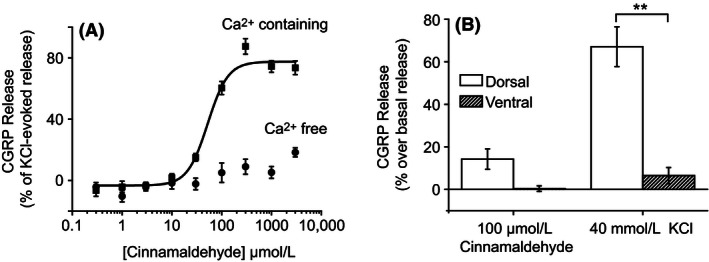
Cinnamaldehyde‐induced calcitonin gene‐related peptide (CGRP) release requires extracellular Ca^2+^ and dorsal but not ventral spinal cord synaptosomes. (A) Cinnamaldehyde‐induced CGRP release from rat spinal cord homogenate was measured by ELISA in the presence and absence of external Ca^2+^. In the absence of external Ca^2+^ ions cinnamaldehyde‐induced CGRP release was attenuated. Representative plot shown, data points are the mean normalized (% of 40 mmol/L KCl‐induced release) release of quadruplicate wells ± SEM. (B) CGRP release from dorsal and ventral spinal cord homogenate was measured by ELISA. Cinnamaldehyde (100 *μ*mol/L)‐induced CGRP release from dorsal spinal cord homogenate (14 ± 5% over basal release; mean ± SEM;* n* = 3 experiments) but did not evoke release from synaptosomal preparations from the ventral spinal cord (0 ± 1% over basal release; mean ± SEM;* n* = 3 experiments). KCl (40 mmol/L) evoked a large increase in CGRP release from dorsal spinal cord homogenate (67 ± 9% over basal release; mean ± SEM;* n* = 3 experiments). The release of CGRP release evoked by KCl was significantly lower in ventral spinal cord homogenate (6 ± 4% over basal release; mean ± SEM;* n* = 3 experiments). Columns represent mean ± SEM (**P < 0.01; *t*‐test *n* = 3 experiments).

In order to examine whether the CGRP release induced by the TRPA1 agonism is dependent on the influx of Ca^2+^ ions, the effect of removing external Ca^2+^ was examined using cinnamaldehyde‐induced CGRP release. In the presence of extracellular Ca^2+^, cinnamaldehyde induced robust increases in CGRP release and in the absence of extracellular Ca^2+^, cinnamaldehyde‐induced CGRP release was attenuated. The average response to 1000 *μ*mol/L cinnamaldehyde in Ca^2+^ free solutions was decreased to 18 ± 6% of the response in Ca^2+^ containing solutions (mean ± SEM; *n* = 3 experiments; Fig. 2). This finding suggests that influx of extracellular Ca^2+^ ions is required for TRPA1 agonist‐induced CGRP release.

### TRPA1‐induced CGRP release requires synaptosomes which originate from the dorsal spinal cord

TRPA1 is thought to be expressed on the nerve terminals of the primary afferent neurons which enter the spinal cord via the dorsal roots and typically terminate within layers I‐V of the dorsal horn (Story et al. 2003). In order to investigate whether the CGRP release evoked by TRPA1 agonists originates from dorsal spinal cord synaptosomes, separate homogenates prepared from the dorsal and ventral spinal cord were stimulated by cinnamaldehyde (100 *μ*mol/L) and KCl (40 mmol/L). Stimulation of dorsal spinal cord homogenate by cinnamaldehyde evoked CGRP release which was on average 14 ± 5% above the basal levels of release (mean ± SEM; *n* = 3 experiments). However, cinnamaldehyde (100 *μ*mol/L) stimulation of ventral spinal cord homogenate failed to evoke CGRP release above basal levels (0 ± 1% over basal release; mean ± SEM; *n* = 3 experiments; Fig. 2). Stimulation of dorsal spinal cord homogenate by KCl (40 mmol/L) evoked large increases in CGRP release which were on average 67 ± 9% over the basal level of release (mean ± SEM; *n* = 3 experiments). However, stimulation of ventral spinal cord homogenate by KCl (40 mmol/L) only evoked a small increase in CGRP release which was significantly lower than the release evoked by dorsal spinal cord homogenate (6 ± 4% over basal release; *P* < 0.01; *t*‐test; *n* = 3 experiments; Fig. 2). These findings suggest that cinnamaldehyde‐evoked CGRP release is dependent on synaptosomes which originate from the dorsal spinal cord. Furthermore, these findings demonstrate that depolarization‐induced CGRP release relies primarily on the release of CGRP from dorsal spinal cord synaptosomes but also indicate that a small amount of CGRP can be released by depolarization of synaptosomes originating from the ventral spinal cord.

### Cinnamaldehyde‐ and AITC‐evoked CGRP release is mediated through activation of TRPA1

**Figure 3:**
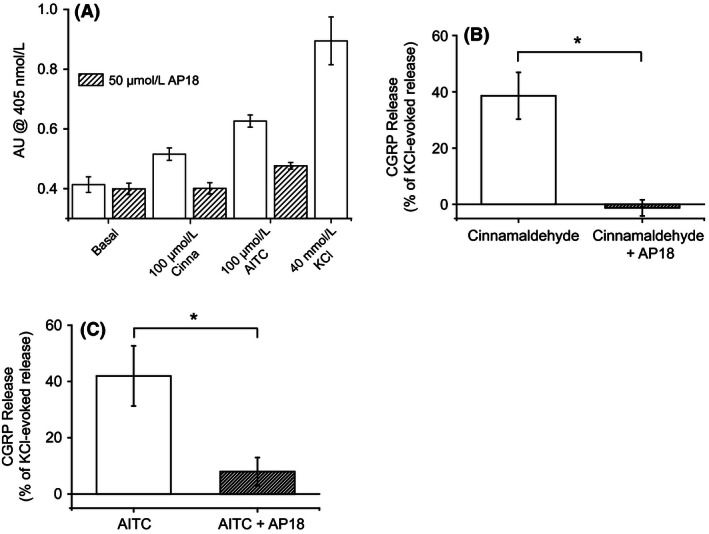
AITC and cinnamaldehyde‐induced calcitonin gene‐related peptide (CGRP) release is inhibited by a transient receptor potential ankyrin 1 (TRPA1) antagonist. The effect of a TRPA1 antagonist, AP18 (50 *μ*mol/L), on cinnamaldehyde (100 *μ*mol/L) and AITC (100 *μ*mol/L) evoked CGRP release (measured by ELISA) from rat dorsal spinal cord homogenate was examined. (A) Graph displaying CGRP release (as measured by absorbance at 405 nmol/L) evoked by 100 *μ*mol/L AITC and 100 *μ*mol/L cinnamaldehyde in the presence and absence of 50 *μ*mol/L AP18 in a representative experiment. Also shown are the levels of CGRP release evoked by negative (basal) and positive (40 mmol/L KCl) controls. Columns represent mean ± SEM of quadruplicate wells. (B) Cinnamaldehyde‐induced CGRP release was significantly inhibited by AP18, release was reduced from 39 ± 8% of 40 mmol/L KCl‐evoked release in the absence of AP18, to −1 ± 3% of 40 mmol/L‐KCl evoked release in the presence of AP18. Columns represent mean ± SEM (*P < 0.05; *t*‐test; *n* = 4 experiments). (C) AITC‐induced CGRP release was also significantly inhibited by AP18, AITC‐induced release was reduced from 42 ± 11% of 40 mmol/L KCl‐evoked release to 8 ± 5% of 40 mmol/L KCl‐evoked release in the presence of AP18. Columns represent mean ± SEM (*P < 0.05; *t*‐test; *n* = 4 experiments).

In order to test whether the CGRP release evoked by TRPA1 agonists is mediated through activation of TRPA1, the effect of a TRPA1 antagonist (AP18) on cinnamaldehyde‐ and AITC‐induced CGRP release was examined. Dorsal spinal cord homogenate was stimulated with 100 *μ*mol/L cinnamaldehyde and 100 *μ*mol/L AITC in the presence and the absence of 50 *μ*mol/L AP18. In the absence of AP18, both cinnamaldehyde and AITC evoked notable increases in CGRP release. Cinnamaldehyde evoked CGRP release (39 ± 8% of 40 mmol/L KCl‐evoked release, mean ± SEM; *n* = 3 experiments) was completely inhibited in the presence of AP18 (average release reduced to −1 ± 3% of 40 mmol/L KCl‐evoked release, mean ± SEM; *P* < 0.05; *t*‐test; *n* = 3 experiments; Fig. 3). Similarly, AITC‐induced CGRP release was significantly inhibited in the presence of AP18, where the average release was reduced from 42 ± 11% to 8 ± 5% of the amount released by 40 mmol/L KCl (mean ± SEM; *P* < 0.05; *t*‐test; *n* = 3 experiments; Fig. 3). Furthermore, AP18 (50 *μ*mol/L) had no effect on CGRP release induced by 40 mmol/L and 60 mmol/L KCl, suggesting that AP18 selectively targets TRPA1. These findings demonstrate that both cinnamaldehyde and AITC evoke CGRP release in this preparation by activating TRPA1.

### VGCC blockade does not affect TRPA1‐induced CGRP release

**Figure 4:**
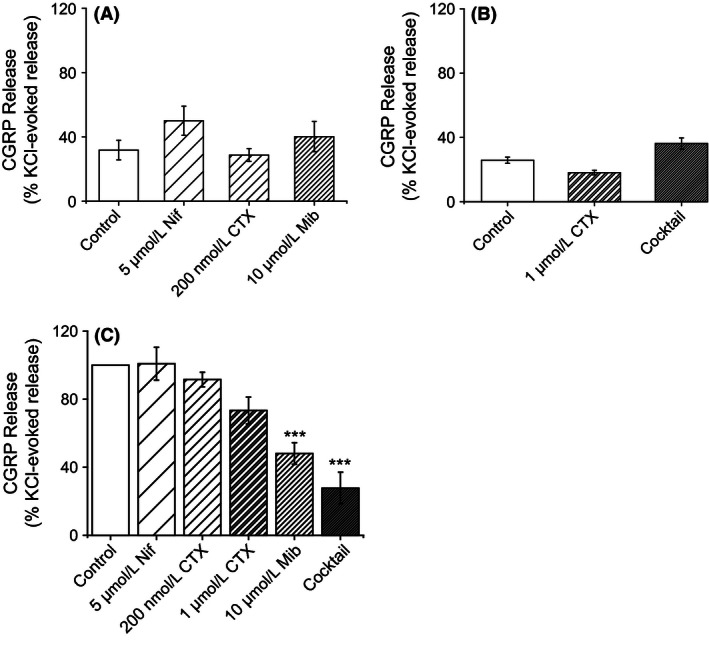
Blocking voltage‐gated calcium channels (VGCCs) inhibits depolarization‐evoked calcitonin gene‐related peptide (CGRP) release but has no effect on cinnamaldehyde‐induced CGRP release. The effect of VGCC‐blockade on 100 *μ*mol/L cinnamaldehyde‐induced CGRP release from rat dorsal spinal cord homogenate was examined. (A) 100 *μ*mol/L cinnamaldehyde‐induced CGRP release (control) was not significantly affected in the presence of 5 *μ*mol/L nifedipine, 200 nmol/L *ω*‐conotoxin MVIIC (CTX) or 10 *μ*mol/L mibefradil dihydrochloride. Columns represent mean ± SEM (ANOVA;* n* = 4 experiments). (B) Moreover, 100 *μ*mol/L cinnamaldehyde‐induced CGRP release (control) was not significantly altered in the presence of 1 *μ*mol/L *ω*‐conotoxin MVIIC (CTX) or a cocktail of VGCC blockers (containing 5 *μ*mol/L nifidepine, 1 *μ*mol/L *ω*‐conotoxin MVIIC and 10 *μ*mol/L mibefradil dihydrochloride). Data are normalized to 40 mmol/L KCl‐induced release. Columns represent mean ± SEM (ANOVA;* n* = 3 experiments). (C) The effect of VGCC‐blockade on depolarization‐induced (40 mmol/L KCl) CGRP release from rat dorsal spinal cord homogenate was also examined. 40 mmol/L KCl evoked CGRP release which was significantly decreased in the presence of the t‐type VGCC blocker, mibefradil dihydrochloride (10 *μ*mol/L) and a cocktail of VGCC inhibitors (containing 5 *μ*mol/L nifidepine, 1 *μ*mol/L *ω*‐conotoxin MVIIC and 10 *μ*mol/L mibefradil dihydrochloride). There was a trend for homogenate treated with 1 *μ*mol/L *ω*‐conotoxin MVIIC (CTX) to exhibit a reduced release to 40 mmol/L KCl although this was not significant. Treatment with 5 *μ*mol/L nifedipine and 200 nmol/L *ω*‐conotoxin MVIIC had no effect on KCl‐induced release. Data is normalized to 40 mmol/L KCl‐induced release (control). Columns represent mean ± SEM (***P < 0.001; ANOVA followed by Tukey's HSD test; nifedipine, 200 nmol/L CTX, 10 *μ*mol/L Mib, *n* = 5 experiments; 1 *μ*mol/L CTX and cocktail, *n* = 4).

TRPA1 channel opening leads to an influx of Na^+^ and Ca^2+^ ions, an event which causes membrane depolarization. The depolarization may, in turn, result in the opening of voltage‐gated ion channels. To investigate whether Ca^2+^ influx through VGCCs contributes to the CGRP release evoked by TRPA1 activation, the effect of VGCC inhibitors on cinnamaldehyde‐induced CGRP release from dorsal spinal cord homogenate was investigated. Dorsal spinal cord homogenate was stimulated with 100 *μ*mol/L cinnamaldehyde in the presence and absence of the L‐type VGCC inhibitor nifedipine (5 *μ*mol/L), the T‐type VGCC inhibitor mibefradil dihydrochloride (10 *μ*mol/L), a low concentration of *ω*‐conotoxin MVIIC (200 nmol/L) to inhibit P/Q‐type VGCCs or a high concentration of *ω*‐conotoxin MVIIC to inhibit N and P/Q‐type VGCCs (1 *μ*mol/L). The CGRP release evoked by 100 *μ*mol/L cinnamaldehyde was not significantly altered in the presence of the VGCC inhibitors (NS; ANOVA; *n* = 3–4 experiments; Fig. 4). However, a trend for increased CGRP release was evident when the homogenate was stimulated by cinnamaldehyde in the presence of nifedipine (5 *μ*mol/L) where release was increased from 32 ± 6% to 50 ± 9% of 40 mmol/L KCl‐evoked release (mean ± SEM; *n* = 4 experiments; Fig. 4).

### T‐type VGCCs are required for CGRP release induced by depolarization

CGRP release from sensory neurons can be evoked by high concentrations of KCl which lead to depolarization and opening of VGCCs. In order to study the differential contribution of VGCC subtypes to depolarization‐induced CGRP release, the effect of VGCC inhibitors on KCl‐induced CGRP release from dorsal spinal cord homogenate was investigated. Dorsal spinal cord homogenate was stimulated with 40 mmol/L KCl in the presence and absence of individual VGCC inhibitors. Depolarization‐induced CGRP release was not affected by the presence of 5 *μ*mol/L nifedipine (L‐type VGCC blockade) or 200 nmol/L *ω*‐conotoxin MVIIC (inhibition of P/Q type VGCCs). However, block of both N and P/Q type VGCCs by 1 *μ*mol/L *ω*‐conotoxin MVIIC lead to a small, but not significant, reduction in depolarization‐induced CGRP release (73 ± 8% of control 40 mmol/L KCl‐evoked release; mean ± SEM; NS; ANOVA followed by Tukey's HSD test; *n* = 4 experiments; Fig. 4). Interestingly, depolarization‐evoked CGRP release was significantly inhibited in the presence of the T‐type VGCC inhibitor, mibefradil dihydrochloride (48 ± 6% of control 40 mM KCl‐evoked release, mean ± SEM; *P* < 0.001; ANOVA followed by Tukey's HSD test; *n* = 5 experiments; Fig. 4). Furthermore, depolarization‐induced CGRP release was profoundly inhibited in the presence a VGCC inhibitor cocktail (containing 5 *μ*mol/L nifidepine, 1 *μ*mol/L *ω*‐conotoxin MVIIC and 10 *μ*mol/L mibefradil dihydrochloride), where CGRP release was reduced to 28 ± 9% of the control 40 mmol/L KCl‐evoked release (mean ± SEM; *P* < 0.001; ANOVA followed by Tukey's HSD test; *n* = 4 experiments; Fig. 4). These findings suggest an importance of T‐ and to a lesser extent N‐type VGCCs for depolarization‐induced CGRP release in this preparation.

### Modulation of depolarization‐induced CGRP release by morphine

**Figure 5:**
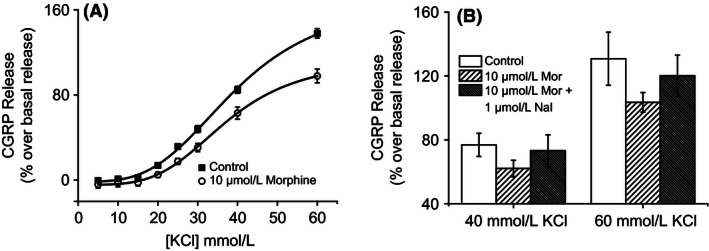
The effect of morphine on depolarization‐induced calcitonin gene‐related peptide (CGRP) release. (A) The effect of morphine on depolarization‐induced CGRP release from rat dorsal spinal cord homogenate was examined. KCl evoked CGRP release in a concentration dependent manner which was inhibited by morphine (10 *μ*mol/L). Representative plot shown, data points are the mean normalized (% over basal) release of quadruplicate wells ± SEM. (B) There was a trend for homogenate treated with morphine to exhibit a reduced CGRP release in response to 40 mmol/L and 60 mmol/L KCl. This effect was reversed in the presence of naloxone. Data are normalized to basal release. Columns represent mean ± SEM (NS; ANOVA followed by Tukey's HSD test; *n* = 4 experiments).

Activation of opioid receptors expressed on sensory neurons causes inhibition of VGCCs. In order to examine whether depolarization‐induced CGRP release could be inhibited by opioid receptor activation, the effects of the prototypical opioid receptor agonist, morphine, on KCl‐induced CGRP release was investigated. Dorsal spinal cord homogenate was stimulated with KCl (5–60 mmol/L) in the presence and absence of 10 *μ*mol/L morphine. A trend for morphine (10 *μ*mol/L) inhibition of CGRP release was observed (Fig. 5A). In the presence of morphine, CGRP release evoked by 40 mmol/L KCl was reduced from 77 ± 7% to 62 ± 5% over basal release (mean ± SEM; NS; ANOVA; *n* = 4 experiments). Furthermore, morphine reduced CGRP release evoked by 60 mmol/L KCl from 131 ± 17% to 103 ± 6% over basal release (mean ± SEM; NS; ANOVA; *n* = 4 experiments). In order to examine whether the inhibitory effects of morphine on CGRP release were mediated by opioid receptors, dorsal spinal cord homogenate was stimulated with KCl (40 and 60 mmol/L) in the presence of morphine (10 *μ*mol/L) and the opioid receptor antagonist naloxone (1 *μ*mol/L). The inhibitory effects of morphine were reduced by naloxone indicating that the release of CGRP can be modulated by opioid receptor activation (Fig. 5B).

## Discussion and Conclusions

This study describes a relatively high‐throughput methodology for examining stimulation of spinal cord synaptosomes and has shown that activation of spinal TRPA1 by pungent food compounds and products of lipid peroxidation evokes CGRP release. Importantly, the results of this study suggest that TRPA1 mediated CGRP release does not require opening of VGCCs. Additionally, the results have demonstrated an involvement of low‐threshold T‐type VGCCs and to a lesser extent, N‐type VGCCs, in the depolarization‐evoked release of CGRP within the spinal cord.

The synaptosomal CGRP release assay allowed full concentration response curves to be constructed for cinnamaldehyde, AITC, 4‐HNE and 4‐ONE. Concentrations of cinnamaldehyde, AITC and 4‐HNE required to evoke CGRP release from synaptosomes were within the same range as those needed to activate TRPA1 channels expressed in recombinant cell systems (Macpherson et al. 2007; Taylor‐Clark et al. 2008; Bandell et al. 2004). However, the concentration of 4‐ONE required for half‐maximal release of CGRP reported in the current study (~64 *μ*mol/L) is higher than a previously reported EC_50_ value (~2 *μ*mol/L) (Taylor‐Clark et al. 2008). AITC has been shown to activate the noxious heat‐sensor TRPV1 at higher concentrations than required to activate TRPA1 (Gees et al. 2013). The contribution of this action to AITC‐induced CGRP release in this preparation is likely to be minimal because both AITC‐ and cinnamaldehyde‐evoked CGRP release was inhibited by the TRPA1‐selective inhibitor, AP18, confirming that the release is mediated by calcium entry through the TRPA1 channel.

CGRP release induced by activation of TRPA1 was restricted to dorsal spinal cord homogenate. Stimulation of ventral spinal cord homogenate with the TRPA1 agonist, cinnamaldehyde, failed to release CGRP above basal levels. In light of the findings that TRPA1 expression in the dorsal spinal cord is abolished with dorsal rhizotomy (Kim et al. 2010), these findings are consistent with TRPA1‐induced CGRP release from the central terminals of TRPA1‐expressing primary afferent neurons which terminate in the superficial layers of the dorsal horn. CGRP release evoked by high potassium was significantly higher in homogenate samples from the dorsal spinal cord in comparison to samples from the ventral spinal cord. However, a small release of CGRP in response to high potassium was evident in ventral spinal cord homogenate; this suggests that depolarization of some motor neuron terminals could lead to the release of CGRP. This finding is consistent with reports of CGRP expression within motor neuron cell bodies of the ventral spinal cord where it is suggested to be involved in repair mechanisms following nerve injury (Chen et al. 2010; McCoy et al. 2012).

Interestingly, inhibition of L‐type, T‐type, P/Q‐type and N‐type VGCCs did not significantly alter the levels of CGRP released by a sub‐maximal concentration of cinnamaldehyde. This finding suggests that Ca^2+^ influx through the TRPA1 channel itself is sufficient to evoke release and is consistent with reports of a high Ca^2+^ permeability of TRPA1 (Zygmunt and Högestätt 2014). Similar findings were reported for TRPV1 in a study examining CGRP release from rat sciatic nerve axons which found that capsaicin‐induced CGRP release was unaffected by blockade of L‐type and T‐type VGCCs both of which were important for high potassium‐induced CGRP release (Spitzer et al. 2008). A trend was observed for potentiation of cinnamaldehyde‐induced release by nifedipine; release was increased from 32 ± 6% to 50 ± 9% (of KCl‐evoked release) in the presence of 5 *μ*mol/L nifedipine. The 1,4‐dihydropyridines have been demonstrated to be TRPA1 agonists and nifedipine has been shown to activate recombinantly‐expressed TRPA1 channels with an EC_50_ value of ~0.4 *μ*mol/L (Fajardo et al. 2008). An increased release of CGRP could therefore be due to the synergistic activation of TRPA1 by cinnamaldehyde and nifedipine, resulting in increased Ca^2+^ influx and CGRP release (Fajardo et al. 2008). Moreover, nifedipine has also been identified as an activator of the sensory neuron‐expressed channel, TRPM3, with an EC_50_ value of ~30 *μ*mol/L (Wagner et al. 2008). Furthermore, a recent study has shown that activation of TRPM3, which is expressed by peptidergic sensory neurons, results in the release of CGRP from mouse skin (Held et al. 2015). Therefore, activation of TRPM3 channels could also contribute to the release of CGRP in this preparation. Activation of TRPA1 or TRPM3 by the L‐type VGCC inhibitor and consequent CGRP release could mask any effects of L‐type VGCC inhibition and makes assessing the role of L‐type VGCCs in cinnamaldehyde‐induced release difficult. However, inhibition of other VGCCs present on sensory nerves had no effect on TRPA1‐induced release, suggesting that TRPA1‐induced CGRP release is largely independent of VGCCs.

In the current study, the contribution of VGCC subtypes to depolarization‐induced CGRP release was also examined. Studies examining CGRP release evoked by high extracellular potassium from isolated lung and skin have identified a reliance upon N‐ and L‐type high VGCC subtypes (Lou et al. 1992; Kress et al. 2001). However, the importance of T‐type low VGCCs was not investigated in these earlier studies. In the current study, high potassium (40 mmol/L) induced CGRP release which was significantly decreased by inhibition of T‐type VGCCs. Mibefradil dihydrochloride, decreased high potassium induced‐CGRP release by ~50%. Consistent with this finding, a dependence on T‐type VGCCs has also been demonstrated for nitric oxide‐induced CGRP release from cultured trigeminal ganglion neurons and high potassium‐induced CGRP release from sciatic nerve axons (Bellamy et al. 2006; Spitzer et al. 2008). Moreover, inhibition of T‐type VGCCs has been shown to decrease mEPSC frequency in the dorsal horn of the spinal cord (Bao et al. 1998). It is important to note that mibefradil dihydrochloride can also inhibit high threshold L‐type VGCC, however, it has a 12–13 fold greater affinity for T‐type VGCCs and the concentration used in this study (10 *μ*mol/L) should act predominantly to inhibit T‐type VGCCs (Martin et al. 2000). Mibefradil has also been reported to inhibit NaN/Nav1.9 and Nav1.8 currents at a concentration of 10 *μ*mol/L (Coste et al. 2007). The contribution of this action to the effect seen with mibefradil dihydrochloride in the current study is uncertain. Interestingly, T‐type calcium channels are implicated in nociception, thermal and mechanical hypersensitivity induced by peripheral nerve injury and diabetic neuropathy has been shown to be alleviated by T‐type channel blockers (Dogrul et al. 2003; Pathirathna et al. 2005; Latham et al. 2009; Lee et al. 2010) and oligodeoxynucleotide antisense knockdown of Cav3.2 (Bourinet et al. 2005).

In contrast to other studies investigating CGRP release (Lou et al. 1992; Kress et al. 2001; Spitzer et al. 2008), pharmacological blockade of L‐type VGCCs had no effect on high potassium‐induced CGRP release. However, as previously discussed, the 1,4‐dihydropyridine drug, nifedipine (5 *μ*mol/L) used to investigate L‐type VGCC involvement can activate TRPA1 within this concentration range and could also activate TRPM3. Such an effect could be masking any potential inhibition of release achieved by block of L‐type VGCCs (Fajardo et al. 2008; Wagner et al. 2008). Interestingly, some studies have used another 1,4‐dihydropyridine compound, nimodipine, which is also an agonist of TRPA1, to test the involvement of L‐type VGCC but reported inhibition of high potassium‐induced CGRP release (Kress et al. 2001; Spitzer et al. 2008).

Pharmacological blockade of P/Q‐type VGCCs alone by a low concentration of *ω*‐conotoxin MVIIC (200 nmol/L) did not significantly alter CGRP release evoked by high potassium. However, block of both N and P/Q type VGCCs by a higher concentration of *ω*‐conotoxin MVIIC (1 *μ*mol/L) lead to a reduction in high potassium‐evoked CGRP release, although this was not a significant effect. This result suggests that N‐type VGCCs could contribute to depolarization‐induced CGRP release. Consistent with this possibility, application of a VGCC inhibitor cocktail reduced high‐potassium evoked release by ~72%, which is a greater inhibition than achieved by mibefradil dihydrochloride alone indicating that other VGCC subtypes are also required for CGRP release.

Opioid receptors are expressed on the peripheral and central terminals of sensory neurons. Activation of opioid receptors causes a decrease in cAMP levels and inhibition of VGCCs (Stein et al. 2003). Consistent with this, morphine inhibited high potassium‐induced CGRP release and this effect could be reversed using the opioid antagonist naloxone. These data are consistent with an anti‐nociceptive effect of morphine in the dorsal horn by presynaptic inhibition of CGRP expressing primary afferent nerve fibers. Similar findings have been demonstrated in a study examining CGRP release from the rat brainstem, which reported that morphine inhibited high‐potassium evoked CGRP release which could be reversed by naloxone (Greco et al. 2014).

In summary, we have shown that activation of TRPA1 channels natively expressed in the rat dorsal spinal cord induces release of the neuropeptide transmitter, CGRP. However, it remains uncertain whether an endogenous TRPA1 agonist would facilitate or inhibit synaptic transmission in the spinal cord. We have demonstrated that TRPA1‐dependent CGRP release does not require activation of T‐type, N‐type or P/Q‐type VGCCs. We have determined that CGRP release evoked by depolarization is predominantly dependent on activation of low‐threshold T‐type VGCCs and can be inhibited by activation of natively‐expressed opioid receptors. This study has demonstrated the use of a dorsal spinal cord homogenate assay for investigation of natively expressed TRPA1 channels and for modulation of depolarizing stimuli at the level of the dorsal spinal cord.

## Author Contributions

T. Quallo, S. Bevan, L. M. Broad, and A. J. Mogg designed the research study. T. Quallo, C. Gentry, and A. J. Mogg performed the research. T. Quallo analyzed the data and wrote the paper.

## Disclosures

The authors declare no conflicts of interest. Eli Lilly does not sell any of the drugs or devices mentioned in this article.

## References

[prp2191-bib-0001] Andersson DA , Gentry C , Moss S , Bevan S (2008). Transient receptor potential a1 is a sensory receptor for multiple products of oxidative stress. J Neurosci 28: 2485–2494. 18322093 10.1523/JNEUROSCI.5369-07.2008 PMC2709206

[prp2191-bib-0002] Andersson DA , Gentry C , Alenmyr L , Killander D , Lewis SE , Andersson A , et al. (2011). TRPA1 mediates spinal antinociception induced by acetaminophen and the cannabinoid Δ(9)‐tetrahydrocannabiorcol. Nat Commun 2: 551. 22109525 10.1038/ncomms1559

[prp2191-bib-0003] Bandell M , Story GM , Hwang SW , Viswanath V , Eid SR , Petrus MJ , et al. (2004). Noxious cold ion channel TRPA1 is activated by pungent compounds and bradykinin. Neuron 41: 849–857. 15046718 10.1016/s0896-6273(04)00150-3

[prp2191-bib-0004] Bao J , Li JJ , Perl ER (1998). Differences in Ca^2+^ channels governing generation of miniature and evoked excitatory synaptic currents in spinal laminae I and II. J Neurosci 18: 8740–8750. 9786981 10.1523/JNEUROSCI.18-21-08740.1998 PMC6793560

[prp2191-bib-0005] Bellamy J , Bowen EJ , Russo AF , Durham PL (2006). Nitric oxide regulation of calcitonin gene‐related peptide gene expression in rat trigeminal ganglia neurons. Eur J Neurosci 23: 2057–2066. 16630053 10.1111/j.1460-9568.2006.04742.x PMC1486900

[prp2191-bib-0006] Bourinet E , Alloui A , Monteil A , Barrère C , Couette B , Poirot O , et al. (2005). Silencing of the Cav3.2 T‐type calcium channel gene in sensory neurons demonstrates its major role in nociception. EMBO J 24: 315–324. 15616581 10.1038/sj.emboj.7600515 PMC545807

[prp2191-bib-0007] Chen L‐J , Zhang F‐G , Li J , Song H‐X , Zhou L‐B , Yao B‐C , et al. (2010). Expression of calcitonin gene‐related peptide in anterior and posterior horns of the spinal cord after brachial plexus injury. J Clin Neurosci 17: 87–91. 19969463 10.1016/j.jocn.2009.03.042

[prp2191-bib-0008] Coste B , Crest M , Delmas P (2007). Pharmacological dissection and distribution of NaN/Nav1.9, T‐type Ca^2+^ currents, and mechanically activated cation currents in different populations of DRG neurons. J Gen Physiol 129: 57–77. 17190903 10.1085/jgp.200609665 PMC2151607

[prp2191-bib-0009] Dogrul A , Gardell LR , Ossipov MH , Tulunay FC , Lai J , Porreca F (2003). Reversal of experimental neuropathic pain by T‐type calcium channel blockers. Pain 105: 159–168. 14499432 10.1016/s0304-3959(03)00177-5

[prp2191-bib-0010] Fajardo O , Meseguer V , Belmonte C , Viana F (2008). TRPA1 channels: novel targets of 1,4‐dihydropyridines. Channels (Austin) 2: 429–438. 18971630 10.4161/chan.2.6.7126

[prp2191-bib-0011] Gangadharan V , Kuner R (2013). Pain hypersensitivity mechanisms at a glance. Dis Model Mech 6: 889–895. 23828645 10.1242/dmm.011502 PMC3701208

[prp2191-bib-0012] Gees M , Alpizar YA , Boonen B , Sanchez A , Everaerts W , Segal A , et al. (2013). Mechanisms of transient receptor potential vanilloid 1 activation and sensitization by allyl isothiocyanate. Mol Pharmacol 84: 325–334. 23757176 10.1124/mol.113.085548

[prp2191-bib-0013] Greco MC , Lisi L , Currò D , Navarra P , Tringali G (2014). Tapentadol inhibits calcitonin gene‐related peptide release from rat brainstem in vitro. Peptides 56: 8–13. 24662320 10.1016/j.peptides.2014.03.009

[prp2191-bib-0014] Held K , Kichko T , Clercq K , De Klaassen H , Bree R Van , Vanherck J‐C , et al. (2015). Activation of TRPM3 by a potent synthetic ligand reveals a role in peptide release. Proc Natl Acad Sci USA 112: 1363–1372. 10.1073/pnas.1419845112 PMC4371942 25733887

[prp2191-bib-0015] Karashima Y , Talavera K , Everaerts W , Janssens A , Kwan KY , Vennekens R , et al. (2009). TRPA1 acts as a cold sensor in vitro and in vivo. Proc Natl Acad Sci USA 106: 1273–1278. 19144922 10.1073/pnas.0808487106 PMC2633575

[prp2191-bib-0016] Kim YS , Son JY , Kim TH , Paik SK , Dai Y , Noguchi K , et al. (2010). Expression of transient receptor potential ankyrin 1 (TRPA1) in the rat trigeminal sensory afferents and spinal dorsal horn. J Comp Neurol 518: 687–698. 20034057 10.1002/cne.22238

[prp2191-bib-0017] Kress M , Izydorczyk I , Kuhn A (2001). N‐ and L‐ but not P/Q‐type calcium channels contribute to neuropeptide release from rat skin in vitro. NeuroReport 12: 867–870. 11277598 10.1097/00001756-200103260-00048

[prp2191-bib-0018] Latham JR , Pathirathna S , Jagodic MM , Choe WJ , Levin ME , Nelson MT , et al. (2009). Selective T‐type calcium channel blockade alleviates hyperalgesia in ob/ob mice. Diabetes 58: 2656–2665. 19651818 10.2337/db08-1763 PMC2768156

[prp2191-bib-0019] Lee MJ , Shin TJ , Lee JE , Choo H , Koh HY , Chung HJ , et al. (2010). KST5468, a new T‐type calcium channel antagonist, has an antinociceptive effect on inflammatory and neuropathic pain models. Pharmacol Biochem Behav 97: 198–204. 20678515 10.1016/j.pbb.2010.07.018

[prp2191-bib-0020] Lou YP , Franco‐Cereceda A , Lundberg JM (1992). Different ion channel mechanisms between low concentrations of capsaicin and high concentrations of capsaicin and nicotine regarding peptide release from pulmonary afferents. Acta Physiol Scand 146: 119–127. 1279940 10.1111/j.1748-1716.1992.tb09399.x

[prp2191-bib-0500] Macpherson LJ , Xiao B , Kwan KY , Petrus MJ , Dubin AE , Hwang S , et al. (2007). An Ion Channel Essential for Sensing Chemical Damage. J. Neurosci. 27: 11412–11415. 17942735 10.1523/JNEUROSCI.3600-07.2007 PMC6673017

[prp2191-bib-0021] Martin RL , Lee JH , Cribbs LL , Perez‐Reyes E , Hanck DA (2000). Mibefradil block of cloned T‐type calcium channels. J Pharmacol Exp Ther 295: 302–308. 10991994

[prp2191-bib-0022] McCoy ES , Taylor‐Blake B , Zylka MJ (2012). CGRP*α*‐expressing sensory neurons respond to stimuli that evoke sensations of pain and itch. PLoS ONE 7: e36355. 22563493 10.1371/journal.pone.0036355 PMC3341357

[prp2191-bib-0023] Mogg A , Mill C , Folly E , Beattie R , Blanco M , Beck J , et al. (2013). Altered pharmacology of native rodent spinal cord TRPV1 after phosphorylation. Br J Pharmacol 168: 1015–1029. 23062150 10.1111/bph.12005 PMC3631388

[prp2191-bib-0024] Pathirathna S , Todorovic SM , Covey DF , Jevtovic‐Todorovic V (2005). 5*α*‐reduced neuroactive steroids alleviate thermal and mechanical hyperalgesia in rats with neuropathic pain. Pain 117: 326–339. 16150542 10.1016/j.pain.2005.06.019

[prp2191-bib-0025] Spitzer MJS , Reeh PW , Sauer SK (2008). Mechanisms of potassium‐ and capsaicin‐induced axonal calcitonin gene‐related peptide release: involvement of L‐ and T‐type calcium channels and TRPV1 but not sodium channels. Neuroscience 151: 836–842. 18178321 10.1016/j.neuroscience.2007.10.030

[prp2191-bib-0026] Stein C , Schäfer M , Machelska H (2003). Attacking pain at its source: new perspectives on opioids. Nat Med 9: 1003–1008. 12894165 10.1038/nm908

[prp2191-bib-0027] Story GM , Peier AM , Reeve AJ , Eid SR , Mosbacher J , Hricik TR , et al. (2003). ANKTM1, a TRP‐like channel expressed in nociceptive neurons, is activated by cold temperatures. Cell 112: 819–829. 12654248 10.1016/s0092-8674(03)00158-2

[prp2191-bib-0028] Taylor‐Clark TE , McAlexander MA , Nassenstein C , Sheardown SA , Wilson S , Thornton J , et al. (2008). Relative contributions of TRPA1 and TRPV1 channels in the activation of vagal bronchopulmonary C‐fibres by the endogenous autacoid 4‐oxononenal. J Physiol 586: 3447–3459. 18499726 10.1113/jphysiol.2008.153585 PMC2538817

[prp2191-bib-0029] Wagner TFJ , Loch S , Lambert S , Straub I , Mannebach S , Mathar I , et al. (2008). Transient receptor potential M3 channels are ionotropic steroid receptors in pancreatic *β* cells. Nat Cell Biol 10: 1421–1430. 18978782 10.1038/ncb1801

[prp2191-bib-0030] Wrigley PJ , Jeong H‐J , Vaughan CW (2009). Primary afferents with TRPM8 and TRPA1 profiles target distinct subpopulations of rat superficial dorsal horn neurones. Br J Pharmacol 157: 371–380. 19371346 10.1111/j.1476-5381.2009.00167.x PMC2707984

[prp2191-bib-0031] Zygmunt PM , Högestätt ED (2014). TRPA1 Pp. 583–630 *in* Nilius B. and Flockerzi V., eds. Mammalian Transient Receptor Potential (TRP) Cation Channels. Springer, Berlin Heidelberg. 25296415

